# Treatment of post-transplantation lymphoproliferative disorders after kidney transplant with rituximab and conversion to m-TOR inhibitor.

**Published:** 2016-12-30

**Authors:** John Fredy Nieto-Rios, Sandra Milena Gómez de los Ríos, Lina María Serna-Higuita, Catalina Ocampo-Kohn, Arbey Aristizabal-Alzate, Kenny Mauricio Gálvez-Cárdenas, Gustavo Adolfo Zuluaga-Valencia

**Affiliations:** 1 Sección Nefrología, Hospital Pablo Tobon Uribe, Medellin,Colombia; 2 Universidad de Antioquia, Medellin, Colombia; 3 Universidad Pontificia Bolivariana, Medellin, Colombia

**Keywords:** Post-transplantation lymphoproliferative disorders, kidney transplantation, rituximab, M-TOR inhibitors

## Abstract

**Background:**

Post-transplantation lymphoproliferative disorders are serious complications of organ transplantation which treatment is not yet standardized.

**Objective:**

To describe the clinical response, overall and graft survival of patients in our center with this complication after kidney transplantation, which received rituximab as part of their treatment as well as conversion to m-TOR.

**Methods:**

Retrospective study, which included patients, diagnosed with post-transplant lymphoproliferative disorders after kidney transplantation from January 2011 to July 2014.

**Results:**

Eight cases were found with a wide spectrum of clinical presentations. Most had monomorphic histology, 85% were associated with Epstein-Barr virus, 25% of patients had tumor involvement of the renal graft, and 12.5% ​​had primary central nervous system lymphoma. All patients were managed with reduction of immunosuppression, conversion to m-TOR (except one who lost the graft at diagnosis) and rituximab-based therapy. The overall response rate was 87.5% (62.5% complete response, 25% partial response). Survival was 87.5% with a median follow-up of 34 months. An additional patient lost the graft, with chronic nephropathy already known. All the remaining patients had stable renal function.

**Conclusions:**

There are no standardized treatment regimens for lymphoproliferative disorders after kidney transplantation, but these patients can be managed successfully with reduction of immunosuppression, conversion to m-TOR and rituximab-based schemes.

## Resumen

**Antecedente:**

La enfermedad linfoproliferativa post-trasplante es una complicación grave del trasplante de órganos cuyo tratamiento aún no se encuentra estandarizado.

**Objetivo:**

Describir la respuesta clínica, supervivencia global y del injerto en pacientes con esta complicación post trasplante renal en nuestro centro y que recibieron rituximab como parte de su tratamiento y la conversión a m-TOR.

**Métodos:**

Estudio retrospectivo que incluyó pacientes con diagnóstico de enfermedad linfoproliferativa postrasplante renal entre enero de 2011 y julio de 2014.

**Resultados:**

Se encontraron ocho casos, con presentaciones clínicas variables. La mayoría correspondieron a histología monomórfica, en 85% se asoció con virus de Epstein-Barr, 25% de los pacientes tenían compromiso tumoral del injerto renal y 12.5% linfoma primario de sistema nervioso central. Todos los pacientes se manejaron con reducción de inmunosupresión, conversión a m-TOR (excepto uno que perdió el injerto al diagnóstico) y tratamiento basado en rituximab. La tasa de respuesta global fue del 87.5% (62.5% respuesta completa, 25% respuesta parcial). La supervivencia fue del 87.5% con una mediana de seguimiento de 34 meses. Un paciente adicional perdió el injerto renal, con nefropatía crónica ya conocida. Los pacientes restantes con función renal estable.

**Conclusiones:**

No existen esquemas estandarizados de tratamiento para la enfermedad linfoproliferativa post-trasplante renal, pero estos pacientes pueden ser manejados de forma exitosa con reducción de la inmunosupresión, conversión a m-TOR y esquemas basados en rituximab.

## Introduction

The first cases of post-transplantation lymphoproliferative disorder (PTLD) were published by Penn *et al.* in 1969 in five patients who received a living donor kidney transplant ^1^; and since then, it remains as one of the complications of higher morbidity and mortality associated with solid organ transplantation.

The term PTLD encompasses a heterogeneous group of lymphoproliferative disorders that may occur after transplantation of solid organs and hematopoietic cells ^2^. Its incidence varies depending on the type of organ transplanted and the type of immunosuppression used; PTLD has been reported in 13%-33% of multivisceral-transplantation recipients, 7%-11% of intestine, 9.4% of heart-lung, 1.8%-7.9% of lung, 3.4% of heart, 2.2% of liver and 1% of kidney ^3^. The current PTLD classification was defined in 2008 by the WHO and is based on the histopathological findings of the tumor ^4^; this classification divides it into four categories: early lesions, monomorphic, polymorphic, and Hodgkin lymphoma.

The non-specific clinical presentation of this disease, together with its broad histopathological spectrum, makes its treatment complex, which can delay the diagnosis and impoverish the prognosis of patients. On the other hand, survival rates are difficult to compare given the broad clinical and histological spectrum, and they additionally depend on the transplanted organ and the localization pattern. For example, Opelz and Döhler in a retrospective study involving 200,000 transplant recipients describe a survival of 65% at 5 years when the organ involved is the allograft, and 22% when the compromise is spread ^5^.

At present, there are no standardized treatments for PTLD due to the low number of cases and the lack of systematic studies. Most of the evidence on which treatment is based comes from case series and retrospective studies ^6^. There is prospective information from phase II studies only for treatment with the anti-CD monoclonal antibody Rituximab ^7^ ^-^ ^9^, and sequential chemotherapy with Rituximab plus cyclophosphamide, doxorubicin, vincristine, prednisone (R-CHOP). Next, we present the experience of our center in the management of this disease with reduction of immunosuppression, conversion to an m-TOR inhibitor (*mammalian target of rapamycin inhibitor*), and treatment based on Rituximab.

## Materials and Methods

Retrospective study performed at the Pablo Tobón Uribe Hospital, Medellín, Colombia. With 372 beds, it is a high complexity center and a referral hospital for a population of 4 million inhabitants. This institution has a multidisciplinary renal transplant group since 2005; approximately 80 renal transplants are performed every year, and 600 renal transplant patients are being followed up; while by outpatient care, 200 patients are treated every month; and in hospital, an average of 60 patients every month, including patients transplanted who come from other institutions.

In this study there were included all renal transplant patients diagnosed with PTLD confirmed by histological findings during the period January 2011 to July 2014; no patient was excluded.

All patients received Rituximab as part of the treatment, and most were converted to m-TOR inhibitors. PTLD was classified according to the World Health Organization criteria for early lesions (plasmacytic hyperplasia, infectious mononucleosis), polymorphic lesions, monomorphic lesions (diffuse B-cell lymphoma, Burkitt's lymphoma, plasmacytoma, plasma cell myeloma, T-cell lymphoma, other) and Hodgkin's lymphoma ^10^. The diagnosis was made by a histopathological analysis of the lesions by an expert in hemato-pathology in all cases; in-situ hybridization was performed in all biopsies to determine the presence of Epstein Barr virus, and the presence of latent membrane protein 1 (LMP-1) was determined by immunohistochemistry. There were also performed extension studies with bone marrow aspirate and biopsy, lactic dehydrogenase, virological studies (Epstein Barr viral load and real-time cytomegalovirus, Elisa for HIV, hepatitis B virus surface antigen, and antibodies to the Hepatitis C virus), contrasted tomography of the skull, neck and thoracoabdominal region; and in some cases, positron emission tomography (PET-CT). The extension of PTLD was determined with the Ann Arbor classification, which divides lymphomas into four stages (I, II, III and IV); it also takes into account the absence or presence of B symptoms, the existence or not of a large tumor mass (greater than 10 cm), splenic and extranodal involvement. A complete remission was defined as the complete disappearance of the neoplasm demonstrated both clinically and by imaging studies. Partial remission was defined as the reduction of more than 50% of tumor mass; and failure to treatment as a reduction of less than 50% of tumor mass, or disease progression. 

All data were obtained from medical records. There were described some demographic data such as age, sex, and clinical data such as the etiology of renal disease; clinical and histological characterization of lymphoproliferative disease; there were described related risk factors for PTLD reported in the literature such as a history of previous rejection diagnosed by renal biopsy, induction therapy used at the time of transplantation, serological status for EBV infection, and immunosuppressive medication. It was evaluated the time elapsed between the transplantation and the diagnosis of PTLD; there were described the treatment used, associated complications, treatment time; and there were evaluated some outcomes in response to treatment, mortality and renal graft survival.

A descriptive analysis of the data was performed, calculating the frequencies and proportions for the qualitative variables; the quantitative variables were described as averages or medians with their respective quartiles. This study was approved by the institution's ethics committee.

## Results

### Demographic findings

Between January 2011 and July 2014, eight patients were diagnosed at our institution with PTLD after renal transplantation. Seven of the patients were recipients of first transplant of deceased donors and one of alive donor (# 4). Seven patients were adults and one pediatric (Table 1). Regarding the serological status for EBV infection, two patients were negative donor, positive recipient; four were positive recipient, positive donor; and one patient was negative recipient, positive donor. Five patients received induction therapy at the time of transplantation (Table 1).

**Table 1:** Baseline characteristics of patients with a diagnosis of Lymphoproliferative Disease associated with renal transplantation

Case	Gender	Age*	Evolution time†	Etiology of the chronic kidney disease	Induction Therapy	History of rejection /Treatment	Epstein Barr Virus Status‡	Cytomegalovirus Status**	Creatinine at diagnosis (mg/dL)
1	Female	55	24	Tubulo-interstitial nephritis by NSAIDs	None	Yes, steroids, Thymoglobulin	(+)	(+)	1.62
2	Male	62	136	Unknown	Daclizumab	No	(+)	(+)	1.05
3	Female	40	11	Focal and segmental glomerulosclerosis	Alemtuzumab	Yes, steroids	(+)	(+)	1.03
4	Male	50	234	Hypertension	No data	Yes, steroids	No data	No data	3.62
5	Female	40	7	Diabetes Mellitus 1	Thymoglobulin	No	(+)	(+)	Loss of graft
6	Male	34	182	Alport's disease	None	No	(-)	(+)	0.94
7	Male	12	9	Posterior Urethra Valves	Thymoglobulin	No	(+)	(+)	0.7
8	Female	69	95	Hypertension	Alemtuzumab	No	(+)	(+)	0.67

*Age at PTLD diagnosis

† Time in months since the trasplant at PTLD diagnosis;

‡IgG receptor, pre-trasplant;

**IgG receptor, pre-trasplant;

 Three patients had at least one acute rejection episode prior to PTLD. Creatinine at the time of diagnosis had a median of 1.03 mg/dL (P25-75 = 0.7-1.6). The median age at diagnosis was 45 years (P25-75 = 23.5-60.3). PTLD presented in four patients during the first two years of transplantation; in one of the patients, it occurred 18 years post renal transplant (# 4).

### Clinical and histological presentation

The clinical presentations were diverse and related to the initial site of tumor involvement, as described in Table 2. All patients had histopathological confirmation; in five or them, it was found monomorphic PTLD (2/5 plasmablastic lymphoma, 3/5 diffuse large B-cell lymphoma); and in two, polymorphic PTLD. In one of the patients (# 2), the diagnosis was made after a lymph node biopsy that reported polymorphic PTLD; however, splenectomy and hepatic biopsy were subsequently defined for non-response, with Hodgkin lymphoma-like PTLD being found in the samples. Six patients were CD20 (+); in contrast, patients with plasmablastic lymphoma diagnosis were CD20 (-). Case 2 had discordant results, the ganglion was CD20 (+) but the spleen and liver samples were CD20 (-). The association with Epstein-Barr virus was confirmed in 6/7 patients (not evaluated in patient # 8) by immunohistochemical staining for latent membrane protein 1 (LMP-1) or by in situ hybridization. In all patients, Epstein-Barr viral load was measured at diagnosis, which was negative in 3 patients (37.5%); during follow-up, this viral load became undetectable in four of them, and in one persisted elevated without being correlated with disease activity. At the time of diagnosis, lactic dehydrogenase was found to be elevated above normal in 50% of patients (4/8) (cases # 1, 2, 5 and 6). Bone marrow involvement was ruled out in all cases.

**Table 2:** Clinical and histological involvement of patients with a diagnosis of lymphoproliferative disease associated with renal transplantation

Case	Stage*	ECOG	International prognostic index†	Compromised organ	Histology‡	Clinical status	CD20	Virus Epstein Barr Virus in tissue	Viral load for Epstein Barr Virus in tissue¶ (copies/mL)
1	It doesn't apply Primary Lymphoma in Central Nervous System	3	2	SNC	Polymorphic	Convulsive syndrome	(+)	(+)**^,^††	28,400 (plasma) 105,960 (LCR)
2	IV	2	4	Spleen, liver, lymph node	Polymorphic (lymph node), Hodgkin, mixed cellularity (spleen, liver)	Febrile syndrome and adenomegalies	Lymph node (+), spleen and liver (-)	(+)**^,^††	31,520
3	I	0	0	Oral cavity	Monomorphic (LBDCG)	Unique mass in oral cavity	(+)	(+)††	Undetectable
4	I	0	0	Oral cavity	Monomorphic (Plasmablastic Lymphoma)	Unique mass in oral cavity	(-)	(+)**	1,816,000
5	IV	3	3	Allograft	Monomorphic (LBDCG)	Renal dysfunction and graft mass	(+)	(+)**	54,160
6	III	3	3	Subcutaneous (thigh)	Monomorphic (Plasmablastic Lymphoma)	Mass in thigh	(-)	(-)**	31,440
7	I	0	0	Allograft	Polymorphic	Febrile syndrome	(+)	(+)**	Undetectable
8	I	2	2	Subcutaneous (neck)	Monomorphic (LBDCG)	Mass in cervical region	(+)	Not performed	Undetectable

*Ann Arbor Classification ^31^

† International prognostic index, low risk (0-1), intermediate low (2), intermediate high (3), high (4-5)

‡Histology according to classification OMS 2008^2^

††Hybridization *in situ*

**LMP-1: latent membrane protein 1,

¶Viral load for Epstein Barr virus by PCR at PTLD diagnosis,

LBDCG: Diffuse large B-cell lymphoma

### Treatment and outcomes

All patients received triple immunosuppressive therapy at the time of diagnosis, as described in Table 3. Subsequent to the diagnosis of PTLD, in all cases it was performed a reduction of immunosuppression (RI), which consisted of the suspension of the anticalcineurinic (7/7), antimetabolite (5/7) and change to mTOR inhibitor (sirolimus n= 4, everolimus n= 3). In one patient (# 5), it was discontinued all immunosuppression following PTLD diagnosis due to graft loss; and it was initiated renal replacement therapy with hemodialysis. The first-line therapy consisted of Rituximab 375 mg/m^2^ body surface area in monitored intravenous infusion for 4 hours, weekly. Patients 3, 4, 6 and 7 received four cycles of Rituximab. Patient 1 received five cycles as the sole therapy; subsequently, the patient received 4 additional cycles in another institution, plus 3200Gys of holoencephalic radiotherapy. One of the patients (# 5), due to the extent of the disease, initially received a course of polychemotherapy with R-CHOP, with partial response. However, it was determined to continue with monotherapy with four cycles of Rituximab with weekly interval, and subsequent therapy with Rituximab (375 mg/m^2)^ with monthly interval with 9 doses. Patient 2 received two cycles of rituximab and was defined as a non-response to treatment, additional diagnostic studies were performed with a splenectomy and liver biopsy, in which it was found Hodgkin lymphoma-like PTLD; therefore the patient received two cycles of ABVD (Adriamycin, bleomycin, vinblastine and dacarbazine) with a partial response to treatment. Table 3 describes the treatments received and the responses of all patients.

**Table 3:** Treatment received, response and evolution of patients with a diagnosis of Lymphoproliferative Disease associated with renal transplantation

Case	Immuno-suppression*	Immuno-suppression†	PTLD Treatment	Tumor Response	Follow-up time (months)	Death
1	Tac,-MMF-Pred	Syr-MMF, pred	Rituximab x 5 weeks	Partial remission	32	No
2	Tac-Aza-Pred	Eve-pred	Rituximab x 2 weeks ABVD x 2	Partial remission	46	No
3	CsA-MMF-Pre	Eve-MMF-Pred	Rituximab x 4 weeks	Complete remission	48	No
4	CsA-MMF-Pre	Syr-Pred	Rituximab x 4 weeks CDE infusion modified x 1	Complete remission, Relapse	17	Yes, secondary to sepsis of pulmonary origin
5	CsA-MMF-Pre	None	Rituximab x 4 weeks + R-CHOP x 1	Partial response	36	No
6	CsA-MMF-Pre	Syr-Pred	Rituximab x 4 weeks	Complete remission, Relapse	70	No
7	Tac-Aza-Pred	Eve-Pred	Rituximab x 4 weeks	Complete remission	32	N
8	CsA-MMF-Pre	Syr-Pred	Rituximab x 4 weeks + CHOP x 4 cycles	Complete remission	28	No

*Immunosuppression at PTLD diagnosis

†Immunosuppression posterior to PTLD diagnosis.

Tac: Tacrolimus, MMF: Mofetil mycophenolate, Aza: Azathioprine, Pred: Prednisolone, CSA: Cyclosporin, Syr: sirolimus, Eve: everolimus, CHOP: Cyclophosphamide, doxorubicin, vincristine, prednisolone, CDE: Cyclophosphamide, doxorubicin (-), etopoxide, ABVD: Doxorubicin, bleomycin, vincristine, dacarbazine.

One patient died during a median follow-up of 34 months (P25-75= 29-47.5 months, minimum 17 months, maximum 70 months). 62.5% of the patients had a complete remission of their disease (# 3, 4, 6, 7, 8); 25.0% had a partial remission (# 1, 5) determined by imaging criteria, however with an excellent clinical response, with recovery of functionality and without B symptoms. Patient number 2 presented a stable disease; ABVD chemotherapy was discontinued after the second cycle as the patient refused to continue because of adverse effects. Patient number 4 presented a pleural, pulmonary and paranasal sinus relapse at 6 months, evidenced by PET-CT, with histological confirmation in the lung; it was performed treatment with CDE chemotherapy (cyclophosfamide, doxorubicin, etoposide) without doxorubicin for advanced heart disease; however, the patient only received one dose due to multiple serious infectious complications leading to death. Patient 6 presented relapse of his disease four years after the diagnosis of PTLD.

### Safety of treatment

Treatment with Rituximab was generally well tolerated; in case 1, pulmonary toxicity was suspected; however, it was the case of a patient with previous pneumonitis due to nitrofurantoin, so the association could not be clearly established; when applying the Naranjo's algorithm to evaluate causality, this yielded a value of three, suggesting a doubtful causality between Rituximab and this adverse effect. Case 3 presented transient leukopenia with a doubtful causality according to the Naranjo's algorithm (score four).

Regarding the evolution of the renal graft, at the end of the follow-up, two patients had graft loss requiring renal replacement therapy; case number 4 was because of chronic graft nephropathy, which progressed, prior to the diagnosis of PTLD; and case number 5 presented PTLD in the renal graft with loss of graft and need for nephrectomy at the time of diagnosis. For the latter patients, serum creatinine values ​​at 6 months and 12 months after the diagnosis of PTLD had a median of 1.1 mg/dL (P25-75= 0.66-1.6) and 1.3 mg/dL (P25-75= 0.75-1.44) respectively. The remaining patients did not present episodes of rejection, despite the reduction of immunosuppression. 

## Discussion

In the present study we report eight cases of post-transplantation renal PTLD that were successfully treated with Rituximab and converted to immunosuppressive therapy with M-TOR. This number of cases did not represent the incidence of PTLD in our group since transplant patients from different institutions were included. Previously we reported a series of 425 patients with renal transplantation between 2005 and 2010, in whom it was predominantly used induction with alemtuzumab (76.2%), finding a low incidence of PTLD (0.47%) ^11^, which was lower than the one reported in literature. Quinlan *et al.* ^12^, published a retrospective cohort study in renal transplant recipients (n = 156,740, years 1999-2007), with a cumulative incidence at 5 and 10 years post-transplant of 0.7% and 1.4%, respectively. An additional study by Caillard *et al.* ^13^, prospectively evaluated new cases of PTLD. The incidence was higher in the first post-transplant year (0.46%, CI 0.32-0.36), with an accumulated incidence after 5 years of 1.18%.

There are several risk factors for PTLD, of which one of the main ones is Epstein-Barr virus (EBV) infection ^14^, which is found in 60% -70% of cases; however, its identification is not necessary for the diagnosis of PTLD ^14^. Other risk factors that have been associated with the development of this disease are the use of inducing drugs such as OKT3 (muronamab) and thymoglobulin, anticalcineurinic agents (cyclosporinee, tacrolimus), other viral infections (cytomegalovirus and hepatitis C), among others (3)(5). Quinlan *et al.* ^12^, found the following as risk factors related to the presence of early PTLD: age under 20 years, non-Hispanic white race and seronegativity for EBV and cytomegalovirus (CMV) at the time of transplantation; and for late PTLD, age under 20 years and over 50, and Hispanic race. In the study by Caillard *et al.* ^13^, age over 60 years at the time of transplantation and receptor seronegativity for EBV were risk factors for the occurrence of PTLD. An additional study also published by Caillard *et al.* ^15^, analyzed a cohort of 25,127 renal transplant recipients, of whom 344 developed PTLD defined as non-Hodgkin's lymphoma (1.4%). They found out that treatment with antithymocyte globulin (AHR = 1.55, 95% CI = 1.20-1.99) and OKT3 (AHR= 1.37, 95% CI= 1.1-1.8), especially when given in the context of rejection, was associated with increased risk of PTLD. Interleukin 2 receptor antagonists (IL-2RA) and sirolimus did not increase the risk. Mycophenolate and azathioprine were associated in this study with a lower risk of PTLD. When immunosuppressants were compared head-to-head in patients without induction, an increased risk of PTLD was found in patients treated with tacrolimus vs. cyclosporine (AHR= 1.57, 95% CI= 1.12-2.19, K= 0.02). In our series, because of the low number of patients, we could not establish any association with known risk factors for the development of PTLD. However, as it was mentioned before, 100% of patients were exposed to anticalcineurinic drugs (n= 3 tacrolimus, n= 5 cyclosporine), two of the patients received induction therapy with thymoglobulin, one additional patient received this in the evolution previous to the diagnosis of PTLD due to a rejection episode, only one patient was seronegative for EBV at the time of transplantation (one without information), and none for CMV; one patient aged over 60 years was transplanted, two under 20 years and none under 10 years.

The clinical behavior and histological appearance of PTLD includes a broad spectrum of presentations. The current histological classification divides the disease into four categories: early lesions, monomorphic, polymorphic and Hodgkin's lymphoma ^4^, with the monomorphic B-cell type being the most common form in more than 70% of cases. The association with EBV is variable but in general is between 60% -70%, and may be up to 100% according to the histological subtype ^14^ ^,^ ^16^, being in our series of 85% (6/7). Among the patients included in this study, the majority had a monomorphic PTLD, of which we present two patients with plasmablastic lymphoma, a rare subtype within this histological category that was initially described in oral cavity in patients with acquired immunodeficiency syndrome (AIDS), but that has also been reported in other immunodeficiencies, including post-transplant and immunocompetent patients ^17^. The location of the neoplasia was similar to that reported in the literature ^13^, with 25% of patients with renal graft involvement and 12.5% ​​with primary central nervous system involvement.

Treatment of PTLD is not standardized. The recommended initial measure is the reduction of immunosuppression, which was performed in 100% of the patients we reported here and which consisted of anticalcineurin suspension in all of them, and antimetabolite in the majority, with change to everolimus or sirolimus. The latter two drugs are m-TOR inhibitors, which exert antineoplastic effects through multiple mechanisms: antiangiogenesis, inhibition of cell replication, inhibition in the production of interleukin-10 (IL-10) and induction of apoptosis ^18^. Although its use has not been evaluated prospectively in patients with PTLD, there are data suggesting a potential role of the conversion to m-TOR inhibitors in the management of this disease following renal transplantation with good results, both in the control of neoplasia and in the functioning of the renal graft ^19^. However, reduction of immunosuppression is not sufficient, with overall response rates in retrospective studies of 45%, high risk of graft dysfunction and early relapse ^20^. The use of chemotherapy has a high probability of success but entails multiple risks of both toxicity and mortality associated with treatment ^21^, which is why Rituximab has been used increasingly frequently in the last decade and is the only treatment that has prospective studies both in monotherapy and in sequential therapy with chemotherapy ^7^ ^-^ ^9^ ^,^ ^22^ ^,^ ^23^.

Rituximab is a chimeric anti-CD20 monoclonal antibody that decreases the number of B lymphocytes; prospective clinical trials have evaluated its efficacy and safety in the treatment of PTLD ^7^ ^,^ ^8^. These studies show that monotherapy with this antibody as a first-line agent after the reduction of immunosuppression is effective, safe, well tolerated and with minimal toxicity ^24^; this drug also has the advantage that it protects the renal graft against rejection by humoral immunity by suppressing CD20+ lymphocytes, which are converted into plasma cells to produce rejection-producing antibodies; the above is very important because the rejection rates in this type of patients are increased by the decrease that is made in immunosuppression. In the series that we report here, no patient presented acute rejection after the reduction of immunosuppression, which we attribute to the strict monitoring that was performed, and the use of Rituximab in all patients.

Since Rituximab monotherapy, especially in patients with extensive disease or with partial remission, the rate of remission is low, with progression and need for additional therapies in up to 50% of cases ^24^ ^,^ ^25^, the most recent proposal found in the literature is to administer a sequential therapy with R-CHOP, with rates of a global response of 90%, but with a high rate of complications and mortality ^23^. In the series reported here, all patients had their immunosuppression decreased, the anticalcineurinic was discontinued, and they were converted to m-TOR and treated with rituximab as specified. Only two patients were treated with R-CHOP with good tolerance. One patient successfully received Rituximab plus whole-brain irradiation for primary central nervous system lymphoma, which has been reported by other authors, despite being known to penetrate poorly in this location ^26^ ^,^ ^27^. The two patients with plasmablastic lymphoma, which by definition is CD20 (-), also received this therapy, and these two patients were the ones who subsequently relapsed from PTLD, indicating that treatment with Rituximab alone probably is not sufficient in this histological variety, nor in patients who are CD20 (-). We did not find in the literature reports of Rituximab treatment for plasmablastic CD 20 (-) lymphoma; the largest series of patients with this type of PTLD has eight patients, in whom a more complete and lasting response was observed with the use of chemotherapy ^28^. 

The prognosis of patients with PTLD is variable among studies. A series published by Ghobrial *et al*. ^29^, which included 107 patients over 33 years, showed an overall response rate of 58% with no difference between patients receiving or not receiving Rituximab (only 27% in this series), which is similar to that reported previously by Leblond *et al* ^30^. Further data published by Evens *et al*. ^31^, suggested improvement in outcomes, especially with the early introduction of Rituximab-based therapies, with overall survival of 73% versus 33% without this drug (p= 0.0001), which supports the growing interest in this type of therapy. In the present series, we describe a survival of 87.5% for a median follow-up of 34 months, with an overall response rate of 87.5%.

The main limitations of our study are the retrospective nature of the study, the low number of patients detected, which did not allow us to evaluate associations with risk factors for disease development or response to treatment, limited follow-up time and biases in individual treatment in the absence of a standardized protocol, as well as the use of different co-interventions, which limits the evaluation of the effect of individual therapies.

In conclusion, although PTLD is a rare complication in renal transplant patients, it can lead to catastrophic consequences in terms of morbidity, mortality and renal graft loss. At present, there are no standardized treatment regimens, but these patients can be treated successfully with reduction of immunosuppression and Rituximab-based regimens, which are well tolerated in patients who have received a solid organ transplant; besides, it has the additional property that protects the patient from rejection. The conversion to m-TOR inhibitor, although not evaluated prospectively, is a strategy that allows complete suspension of the anticalcineurinic, protecting the function of the transplanted organ. It is important, with the current evidence and available prospective studies, to design and propose a protocolized management strategy for this type of patients.
